# Ti_3_AlC_2_ MXene nanosheets as a novel corrosion inhibitor for carbon steel in 0.5 M sulfuric acid solution†

**DOI:** 10.1039/d3ra07341f

**Published:** 2024-01-31

**Authors:** Xuehong Min, Shiquan Ma, Zongyi Zhou, Dequan Wu, Bokai Liao

**Affiliations:** a Wuhan Business University Wuhan 430056 P. R. China; b School of Chemistry and Chemical Engineering, Guangzhou University Guangzhou 510006 P. R. China bokailiao@gzhu.edu.cn; c Southwest Institute of Technology and Engineering Chongqing 400039 P. R. China Wu600527@yeah.net; d Joint Institute of Guangzhou University & Institute of Corrosion Science and Technology, Guangzhou University Guangzhou 510006 P. R. China

## Abstract

Herein we report that Ti_3_AlC_2_ MXene nanosheets were identified as a highly effective cathodic protection corrosion inhibitor for carbon steel in hydrochloric acid solution. Ti_3_AlC_2_ Mxene nanosheets form a stable inhibition layer on metal surfaces due to their high adsorption capacity and act as a barrier or protective film to prevent attacks from corrosive substances and thus lead to an extended metal service life.

## Abstract 2

Herein, anti-corrosion behavior and mechanism of Ti_3_AlC_2_ MXene nanosheets were firstly studied, which formed the protective film on metal surface due to its high adsorption capacity.

## Graphical abstract

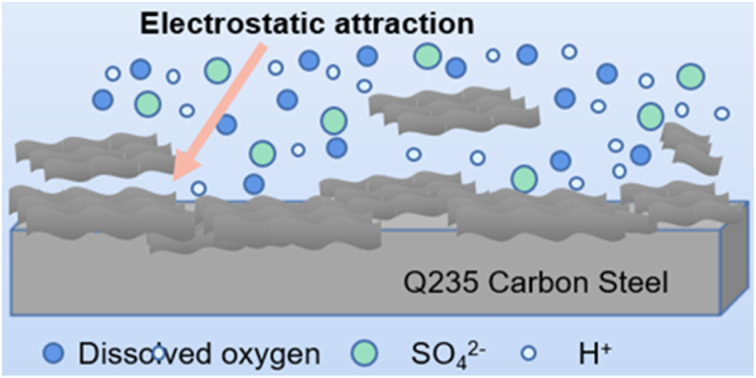

Corrosion is a spontaneous phenomenon for most metal materials, which results in loss of resources and expensive maintenance. The estimated cost of corrosion is about 3–5% of the gross domestic product (GDP) for most countries.^1^ Developing an effective corrosion-control method such as addition of corrosion inhibitors, anti-corrosion coating, corrosion-resistance alloys, sacrificial anodes, and cathodic protection, is of great importance for sustainable development.^2–4^ The corrosion inhibitor method is a cost-effective and efficient solution to control corrosion, which is widely used in fields such as the petroleum and gas industry, automobile manufacturing, water systems, and pickling. Traditional inhibitors often contain hazardous components such as heavy metal ions, organophosphates, and amines, posing a significant environmental and human health threat. Consequently, eco-friendly corrosion inhibitors have garnered considerable attention in recent years.

Lamellar MXene is a highly regarded 2D material with high thermal and electrical conductivities, excellent flexibility, and good electron mobility, which has potential applications in wearable devices and flexible electronics.^5,6^ The chemical formula for MXene materials is M_*n*+1_X_*n*_T_*x*_, where M denotes a transition metal, *n* ranges from 1 to 3, X signifies C and/or N elements, and T_*x*_ represents surface functional groups, including F^−^, OH^−^, or O^2−^. The preparation of Ti_3_AlC_2_ MXene nanosheets by the process of selectively etching MAX-phase materials using a potent acid makes them rich in surface-terminating groups. For example, MXene nanosheets can be incorporated into epoxy coatings to serve as a physical barrier, elevating its anti-corrosion capacity.^7–9^ Yan *et al.*^10^ prepared multiple layers of Ti_3_AlC_2_ MXene nanosheets by etching the aluminum atomic layer of the Ti_3_AlC_2_ precursor and then adding them to the epoxy coatings, acting as a physical barrier. Zhou *et al.*^11^ utilized 2D MXene nanosheets in a reversible self-healing coating based on the Diels–Alder reaction to achieve light-induced self-healing performance. To date, the application of Ti_3_AlC_2_ MXene nanosheets as corrosion inhibitors for enhancing metal anti-corrosion performance has not been reported. In this work, Ti_3_AlC_2_ MXene nanosheets were prepared and applied as a novel and highly efficient corrosion inhibitor for carbon steel in an acidic medium, and the relevant anti-corrosion mechanism was proposed.

As shown in Fig. 1a, the prepared Ti_3_AlC_2_ MXene nanosheets showed a two-dimensional layered microstructure. The zeta potential of Ti_3_AlC_2_ MXene nanosheets reached −45.1 mV, indicating that Ti_3_AlC_2_ MXene nanosheets had good attraction and could be adsorbed and gathered on the metal surface to form protective films. The zeta potential value below −30 mV or above +30 mV results in strong repulsive forces and improves the physical colloidal stability. Aggregation of emulsion droplets or colloids occurs if the zeta potential falls below these levels owing to attractive forces.^12–14^Fig. 1d_1_ shows the equivalent circuit diagram of the blank group, and Fig. 1d_2_ shows the equivalent circuit diagram of adding the MXene corrosion inhibitor group. Fig. 1(c–g) shows the Nyquist and Bode plots for EIS measurements of carbon steel in 0.5 M H_2_SO_4_ solution containing various MXene inhibitor concentrations at 298 K.^15^ An increase in the inhibitor concentration leads to the increase of modulus in Bode plot and an extended semicircle in the Nyquist plot. As shown in Fig. 1e, because the peak value of the Nyquist plot is shifted to the right, the peak became wider overall, which could be due to the overlap of the two peaks. Thus, a two-time-constant equivalent circuit was introduced in this study after Mxene as a corrosion inhibitor was added. The equivalent circuit model diagram presented in Fig. 1 d_1_ and d_2_ reflect the electrical properties of the metal/solution interface, and the fitting parameters are listed in Table S2.† The value of *R*_ct_ increased from 7.94 Ω cm^2^ (blank) to 381.6 Ω cm^2^ (30 mg L^−1^), 670.1 Ω cm^2^ (50 mg L^−1^), 1179 Ω cm^2^ (100 mg L^−1^) and 1284 Ω cm^2^ (200 mg L^−1^). Moreover, the highest corrosion inhibition efficiency reached 99.38%. The decrease of the double layer capacitance value indicates the replacement of the water molecule by the inhibitor, enlarging the thickness of the double layer. These findings illustrate that the use of MXene inhibitors can considerably reduce the rate of corrosion for mild steel under acidic conditions.^16^Fig. 1g shows the polarization curves used to study carbon steel corrosion in 0.5 M H_2_SO_4_ at varying inhibitor concentrations; the fitting parameters are shown in Table S3.† The corrosion current density was 1.64 × 10^−3^ A cm^−2^ (blank) > 4.75 × 10^−5^ A cm^−2^ (30 mg L^−1^) > 4.05 × 10^−5^ A cm^−2^ (50 mg L^−1^) > 3.91 × 10^−5^ A cm^−2^ (100 mg L^−1^) > 3.12 × 10^−5^ A cm^−2^ (200 mg L^−1^), in which 200 mg L^−1^ MXene solution reached the highest corrosion inhibition efficiency (*η*, 98.09%).^17–19^ Moreover, the corrosion potential shifted to a positive direction and the potential difference was larger than +85 mV, indicating that the MXene inhibitor belonged to the anode-dominant type corrosion inhibitor. The concentration of MXene nanosheets affects the adsorption on the steel surface, forming a dense resistance layer that protects against further evasion in acidic media.^20^Fig. 1h depicts the change of corrosion inhibition efficiency with inhibitor concentration based on weight loss (Table S1†) and electrochemical results. The tendency was basically consistent, and increasing inhibitor concentration could enhance the corrosion inhibition efficiency, indicating its potential for preventing corrosion and extending equipment lifespan.

**Fig. 1:**
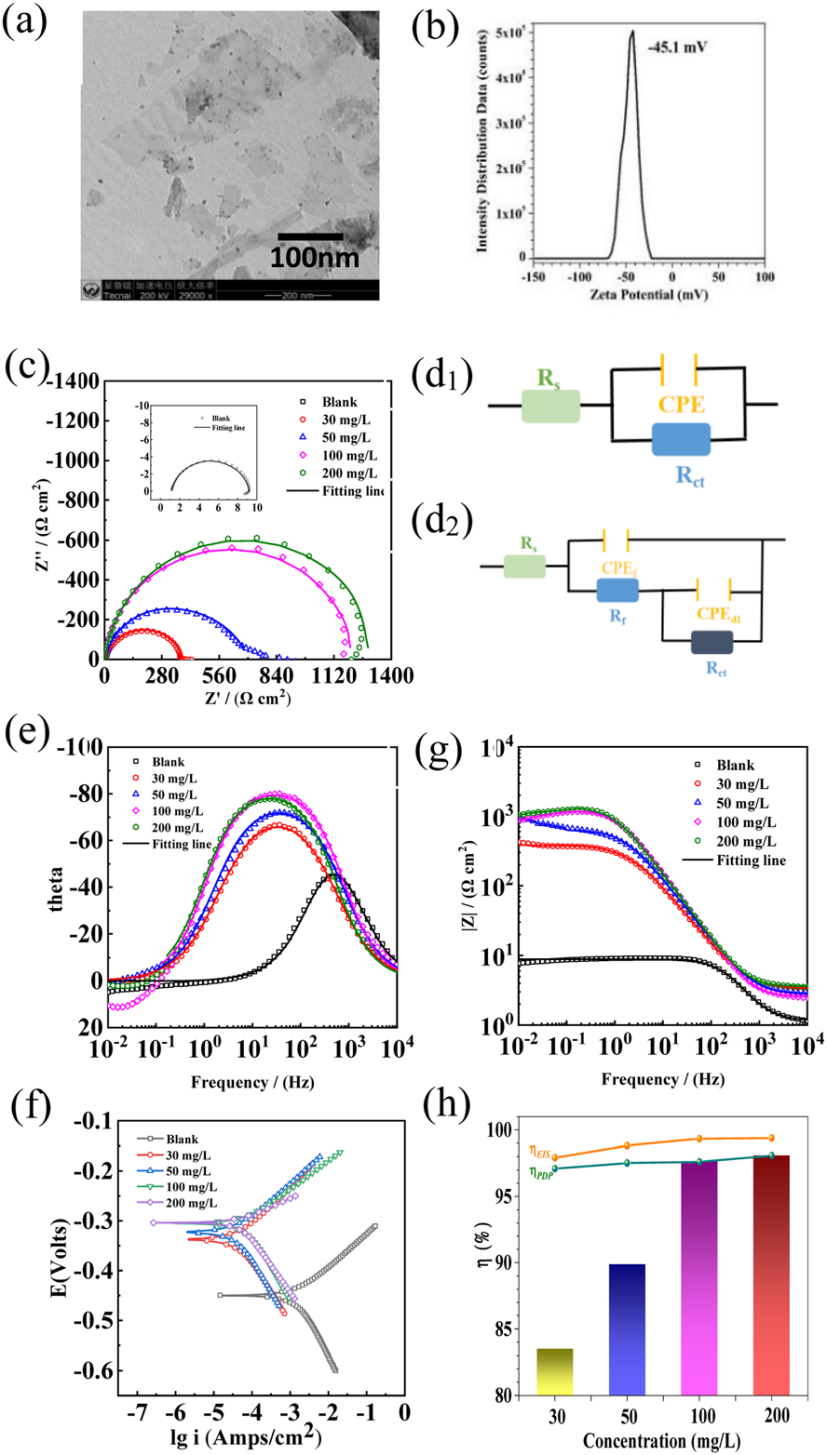
SEM (a) zeta potential (b), EIS (c, e and g), equivalent circuit diagrams (d_1_ and d_2_), PDP (f) and weight loss efficiency diagram (h) of Q235 in the presence of different concentrations of MXene inhibitor added in 0.5 M H_2_SO_4_ for 0.5 h.

The real-time EIS test was carried out to monitor the long-term anti-corrosion performance of the inhibitor, as shown in Fig. S1.† As shown in Fig. 2, in the absence of an inhibitor, *R*_ct_ decreased with immersion time, indicating that the corrosion rate increased with time in the blank solution.^21^ For example, the value of *R*_ct_ decreased from 10.99 Ω cm^2^ for 24 h to 7.15 Ω cm^2^ for 144 h. With the addition of MXene inhibitor, *R*_ct_ increased greatly compared to that in the blank solution and it increased with the increasing addition amount, suggesting that the metal corrosion rate was significantly reduced. For example, *R*_ct_ increased to 1302 Ω cm^2^ in the presence of 200 mg L^−1^ MXene inhibitor at 24 h. With the prolonged immersion time, *R*_ct_ and the corresponding corrosion inhibition efficiency *η*_EIS_ of MXene inhibitor slightly decreased but could remain at a high level. For example, after 144 hours immersion, the inhibition efficiency of 200 mg L^−1^ MXene inhibitor was found to be 98.98%, demonstrating its superior long-term persistence. Compared with the addition of 30 mg L^−1^ inhibitor, the downward trend decreased with increasing concentration.

**Fig. 2:**
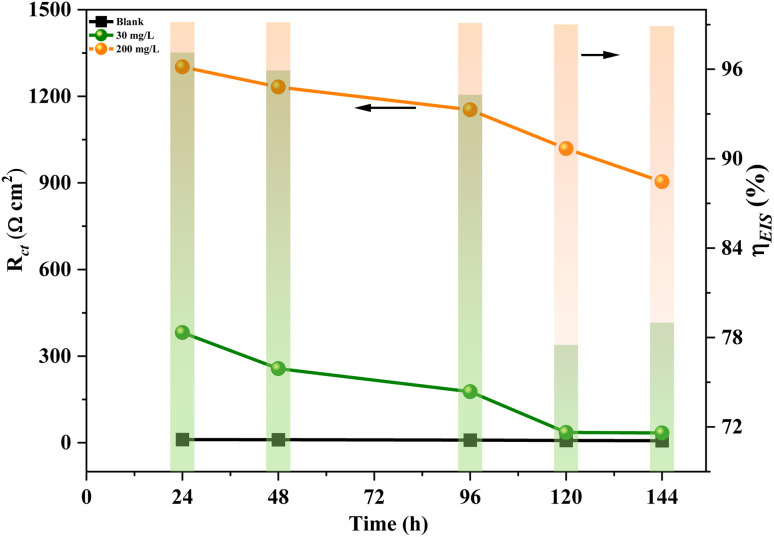
Change of *R*_ct_ and *η*_EIS_ for Q235 carbon steel after immersion in 0.5 M H_2_SO_4_ solution with varying concentrations (0, 30, and 200 mg L^−1^) of MXene.

Fig. 3 shows the SVET diagrams of carbon steel in 0.5 M H_2_SO_4_ solution without and with MXene corrosion inhibitor for 0 h and 24 h. As shown in Fig. 3a, the region in orange or red color presents an anodic dissolve site while the blue or purple region means the cathodic site. After immersion for 24 h in a blank (Fig. 3b), the area and intensity of anodic sites became larger, suggesting the anodic dissolution rate was increased. With the addition of 200 mg L^−1^ inhibitor, the area and intensity of the anodic sites became smaller, meaning MXene inhibitor can effectively retard the anodic dissolution process. In summary, the inhibition mechanism of Ti_3_AlC_2_ MXene nanosheets on carbon steel in an acidic electrolyte could be drawn as follows: the modified-layered MXene remained negatively charged and dispersed in water; it was attracted to iron ions in its nearest neighbours. The matched polarity allowed MXene to migrate towards the surface of the carbon steel, which was further facilitated by the electron sharing between the titanium and iron atoms on the MXene.^22^ As a result, MXene nanosheets tightly covered the corrosion-sensitive sites and acted as a barrier against the participation of corrosive media in corrosion.^23–25^ Therefore, Ti_3_AlC_2_ MXene nanosheets had a superior barrier effect, explaining the protective mechanism of its protection.

**Fig. 3:**
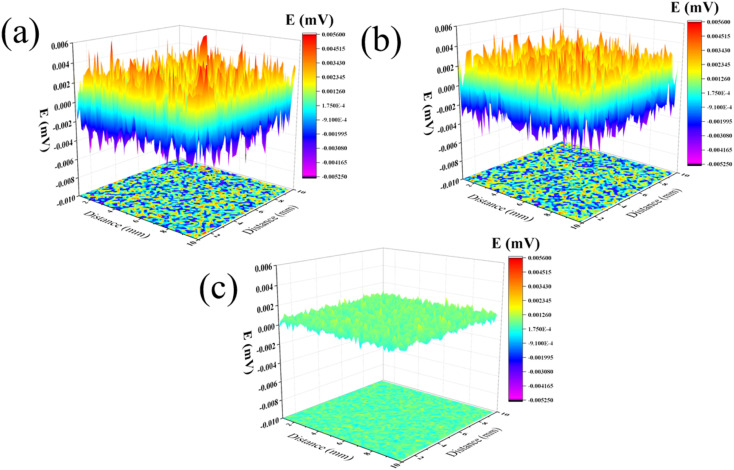
Q235 in a 0.5 M H_2_SO_4_ solution with and without 200 mg L^−1^ MXene inhibitor after different immersion times: 0 h (a, initial state), 24 h (b, without inhibitor), and 24 h (c, with inhibitor).

In summary, the Ti_3_AlC_2_ MXene nanosheets, a novel nanomaterial, served as a corrosion inhibitor for carbon steel in sulfuric acid solution for the first time. Results confirmed that Ti_3_AlC_2_ MXene nanosheets could protect carbon steel effectively and the inhibition efficiencies reached 98.98% even at 200 mg L^−1^. Comprehensive measurements and characterizations were used to study the inhibition mechanism of Ti_3_AlC_2_ MXene nanosheets.

## Conflicts of interest

The authors declare no competing financial interest.

## Supplementary Material

RA-014-D3RA07341F-s001

## References

[cit1] Li X., Zhang D., Liu Z., Li Z., Du C., Dong C. (2015). Materials science: Share corrosion data. Nature.

[cit2] Angst U. M. (2019). Corros..

[cit3] Cui S., Wang J., Mi L., Chen K., Ai W., Zhai L., Guan X., Zheng Y., Wang D. (2022). Nano Energy.

[cit4] Wan S., Chen H., Liao B., Guo X. (2024). Prog. Org. Coat..

[cit5] Lu J., Jia P., Liao C., Xu Z., Chu F., Zhou M., Yu B., Wang B., Song L. (2022). Composites, Part B.

[cit6] Malaki M., Varma R. (2023). Nano-Micro Lett..

[cit7] Abdossamadi S., Rabbani-Chadegani A., Shahhoseini M. (2014). J. Biomol. Struct. Dyn..

[cit8] Kannan P., Rao T., Rajendran N. (2018). J. Colloid Interface Sci..

[cit9] Fiori-Bimbi M., Alvarez P. E., Vaca H., Gervasi C. A. (2015). Corros. Sci..

[cit10] Yan H., Li W., Li H., Fan X., Zhu M. (2019). Prog. Org. Coat..

[cit11] Wang Z., Liang H., Yang H., Xiong L., Zhou J., Huang S., Zhao C., Zhong J., Fan X. (2019). Prog. Org. Coat..

[cit12] Raja P. B., Ismail M., Ghoreishiamiri S., Mirza J., Ismail M. C., Kakooei S., Rahim A. A. (2016). Chem. Eng. Commun..

[cit13] Li Y., Cheng Y. F. (2016). Appl. Surf. Sci..

[cit14] Zehra B. F., Said A., Eddine H. M., Hamid E., Najat H., Rachid N., Toumert L. I. (2022). J. Mol. Struct..

[cit15] Taghavikish M., Dutta N., Roy Choudhury N. (2017). Coatings.

[cit16] Dindodi N., Shetty A. N. (2019). Arabian J. Chem..

[cit17] Wang N., Mu Y., Xiong W., Zhang J., Li Q., Shi Z. (2018). Corros. Sci..

[cit18] Aghaaminiha M., Mehrani R., Colahan M., Brown B., Singer M., Nesic S., Vargas S. M., Sharma S. (2021). Corros. Sci..

[cit19] Zheng H., Zhang B., Wang X., Lu Y., Li F., Li C. (2023). Chem. Eng. J..

[cit20] Yan L., Xu Z., Deng N. (2019). Prog. Org. Coat..

[cit21] Oyewole O., Oshin T. A., Atotuoma B. O. (2021). Heliyon.

[cit22] de Oliveira M. C. L., da Silva R. M. P., Souto R. M., Antunes R. A. (2022). J. Magnesium Alloys.

[cit23] Díaz-Cardenas M. Y., Valladares-Cisneros M. G., Lagunas-Rivera S., Salinas-Bravo V. M., Lopez-Sesenes R., Gonzalez-Rodríguez J. G. (2017). Green Chem. Lett. Rev..

[cit24] Ge S., Hu P., Deng J., Li S., Xing H., Han J., Hua X., Wang L., Yang J., Jin B., Zhang W., Wang K. (2023). Tungsten.

[cit25] Luo Z., Zhang Y., Wang H., Song L., Liao B., Guo X. (2024). Corros. Sci..

