# Supplementary material for: Ti3AlC2 MXene nanosheets as a novel corrosion inhibitor for carbon steel in 0.5 M sulfuric acid solution
Source: RSC Adv. 2024 Jan 31;14(7):4335–8. doi: 10.1039/d3ra07341f (PMC10828932; doi:10.1039/d3ra07341f)
Supplement: RA-014-D3RA07341F-s001 [file RA-014-D3RA07341F-s001.pdf]

Electronic Supplementary Material (ESI) for RSC Advances.
This journal is © The Royal Society of Chemistry 2024

Electronic Supplementary Information

Ti_3AlC_2 MXene nanosheets as novel corrosion inhibition for carbon steel in 0.5 M sulfuric acid solution

Xuehong Min,‡[a] Shiquan Ma,‡[b], Zongyi Zhou[b], Dequan Wu[c]* and Bokai Liao[b, d]*

[a] Equestrian College, Wuhan Business University, Wuhan, 430056, P. R. China;

[b] School of Chemistry and Chemical Engineering, Guangzhou University, Guangzhou, 510006, P. R. China;Equine Science Research and Doping Control Center, Sports School • International

[c]Southwest Institute of Technology and Engineering, Chongqing, 400039, P. R. China;

[d]Joint Institute of Guangzhou University & Institute of Corrosion Science and Technology, Guangzhou University, Guangzhou, 510006, P. R. China;

E-mail: Wu600527@yeah.net, bokailiao@gzhu.edu.cn.

1. Experimental Section

1.1 Materials

Ti_3AlC_2 nanosheets were synthesized by etching Ti_3C_2 phase with LiF/HCl as described earlier. The process involves adding Ti_3C_2 phase to 10 mL of 9 M HCl containing 1.0 g of LiF and 0.16 g of $AlCl_3$, followed by 3 days of etching at room temperature. The precipitate obtained was washed several times with deionized water until neutral pH was reached and dispersed in TMAOH (25% wt in water) for 3 days with stirring. After centrifugation and repeated washing with deionized water and ethanol, the collected precipitates were re-dispersed in water using sonication in a water bath. Finally, an aqueous solution of Ti_3AlC_2 nanosheets were obtained from the supernatant after centrifugation at 7000 rpm. The morphology and microstructure of Ti_3AlC_2 nanosheets were investigated by scanning electron microscope (SEM, SU1510, Japan). The Zeta potential technique was carried out to determine the stability and charge of nanoparticles.

The powder form of Ti_3AlC_2 (200-mesh) and TMAOH solution with a concentration of 25% in water were acquired from Shanghai Macklin Biochemical Co., Ltd. High-purity HCl solution (> 98%) with a concentration of 36% (w/w) and $AlCl_3$ were obtained from Sinopharm Chemical Reagent Co., Ltd. The study used ultrapure water with a resistivity of 18.2 MΩ·cm sourced from Milli-Q Biocel. Q235 carbon steel with 1 × 1× 0.5 mm was well polished using SiC paper with 180 to 1500 mesh before each weight and electrochemical tests.

1.2 Measurement of corrosion rate and corrosion inhibition efficiency

The polished Q235 carbon steel sample was weighed at least three times by analytical balance and then averaged before and after being soaked in H_2SO_4 solution containing different concentrations of MXene corrosion inhibitor at room temperature. The surface of sample was cleaned using the pickling solution containing 1%

methenamine. Corrosion rate and corrosion inhibition efficiency (η) can be calculated as follows:

$$v = \frac{w_0 - w}{S_t} \times 100\% \quad (1)$$

$$\eta = \frac{v_0 - v}{v_0} \times 100\% \quad (2)$$

Where, w_0 and w are the mass loss of Q235 carbon steel without and with MXene inhibitor, v_0 and v are the corresponding average corrosion rate without and with corrosion inhibitor, t is the immersion time, and S_t is the exposed electrode area.

1.3 Measurement of electrochemical impedance spectra (EIS) and potentiodynamic polarization curves (PDP)

The CS350 electrochemical workstation (Corrtest, China) with a three-electrode configuration was used to measure electrochemical impedance spectra (EIS) and potentiodynamic polarization curves (PDP) for evaluation of anti-corrosion performance of Ti_3AlC_2 MXene nanosheets. The encapsulated carbon steel with exposed area 1 cm^2 was working electrode, saturated calomel electrode (SCE) acted as reference electrode and platinum tablet with exposed area 2 cm^2 acted as counter electrode, respectively. PDP tests were carried out with a scanning rate of 0.5 mV/s ranging from -0.15 to +0.15 V versus open circuit potential. Tafel extrapolation was uniformly employed to fit the polarization data. The region on the polarization curve that conformed to the Tafel relationship was selected, namely, the area where a linear relationship existed between current density and electrode potential. Within the designated Tafel region, a linear regression method was applied to fit the data. The slope of the fitted line represented the Tafel slope, while the intercept could be used to calculate the corrosion current density. The physicochemical meanings of each parameter for the polarization curve were as follows: I_0 was corrosion current density and E_{corr} was self-corrosion potential. η_{PDP} is the anti-corrosion effectiveness from PDP method could be calculated as Eq. (3).

$$\eta_{PDP} = \frac{I_0 - I_0^{'}}{I_0} \times 100\% \quad (3)$$

where I_0 and $I_0^{'}$ were the corrosion current density of Q235 carbon steel without and with corrosion inhibitor, respectively.

EIS tests employed a sinusoidal wave disturbance of 10 mV amplitude at frequency range of 10^4 to 10^{-2} Hz. The physical and chemical meanings of each parameter in the equivalent circuit in the following table were: R_s was solution resistance. CPE_f and CPE_{dl} were the double layer capacitor of film and electric double layer, respectively. R_f was the resistance of film and R_{ct} was the resistance due to charge Transfer. The η_{EIS} was calculated as follows:

$$\eta_{EIS} = \frac{R_{ct} - R_{ct}^0}{R_{ct}} \times 100\% \quad (4)$$

where R_{ct} and R_{ct}^0 respectively represented the charge transfer resistance in the presence and absence of MXene corrosion inhibitor, respectively.

The variance (σ) was displayed to illustrate the accuracy of fitting process. According to the principle of a parallel-plate capacitor, capacitance was proportional to the plate area and inversely proportional to the thickness of the insulator. This relationship was expressed as Eq. (5):

$$C = \frac{\varepsilon S}{d} \quad (5)$$

where ε was the dielectric constant, d was the thickness of the insulator, and S represented the plate area.

The SVET measurement technique was employed to investigate the localized corrosion behavior using the VersaSCAN microelectrochemical system. The vibration amplitude was 30 μm and swept area was set as 6 × 6 mm^2.

2. Supplementary Figures

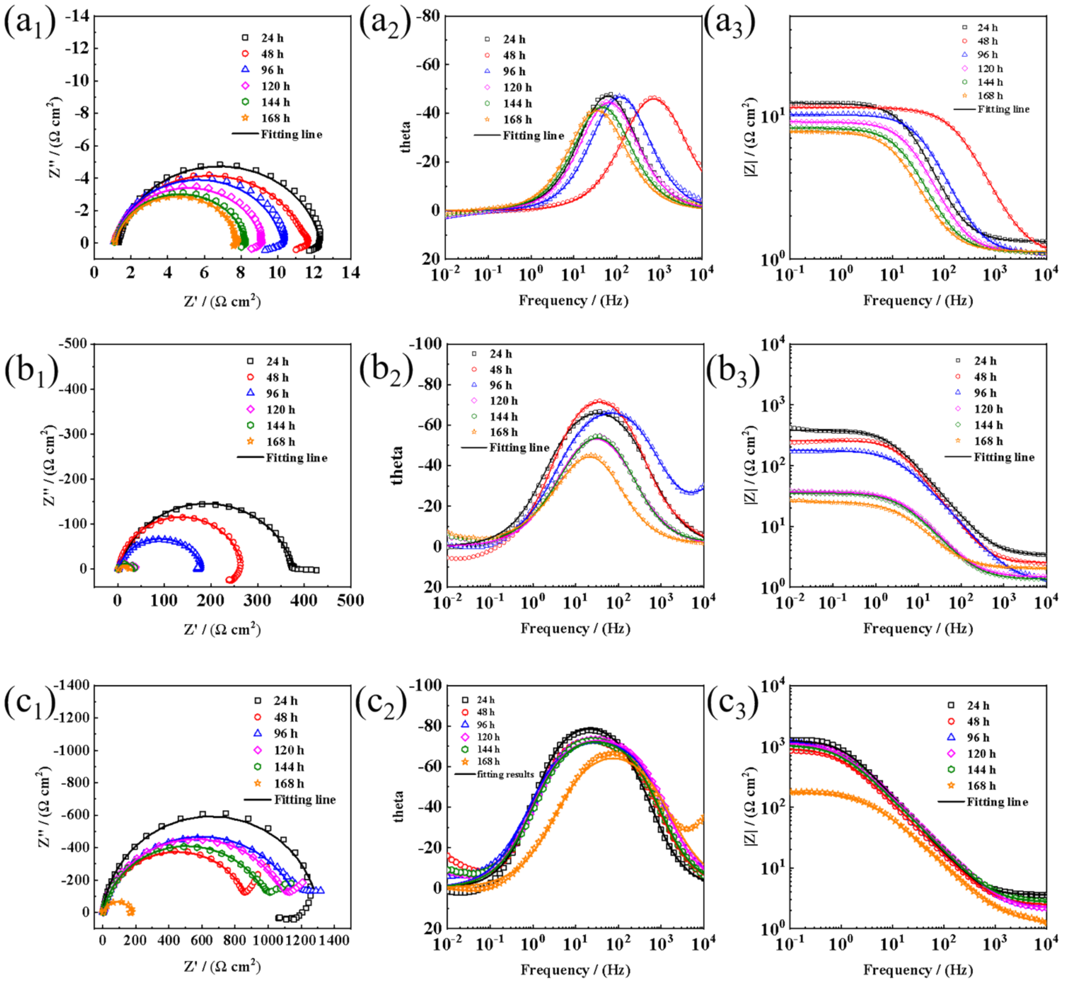

Fig. S1 Variations of EIS plots versus time for Q235 carbon steel immersing in 0.5 M H_2SO_4 solution under different concentration of Mxene nanosheets: (a_1-a_3), 0 mg/L (b_1-b_3), 30 mg/L (c_1-c_3) 200 mg/L.

C_{MXene} (mg/L)	ΔW (g)	Corrosion rate ($g \cdot m^{-2} \cdot h^{-1}$)	η (%)
0	0.1738	0.72417	——
30	0.0287	0.11958	83.48
50	0.0176	0.07333	89.87
100	0.0041	0.01708	97.64
200	0.0033	0.01375	98.10

Table S1 Parameters of weight loss tests for Q235 carbon steel with different concentrations of Mxene nanosheets.

Concentration (mg/L)	R_s ($\Omega\cdot cm^2$)	CPE_f ($S^{n1}\cdot\Omega^{-1}\cdot cm^2$)	R_f	CPE_{dl}	R_{ct} ($\Omega\cdot cm^2$)	η_{EIS} (%)	σ
0	1.15	2.23×10^{-4}	-	-	7.94±1.10	——	1.73×10^{-3}
30	3.36	3.11×10^{-4}	372.5	0.91	381.6±0.15	97.92±0.12	4.16×10^{-3}
50	2.76	2.11×10^{-4}	608.5	0.82	670.1±12.05	98.82±0.19	6.53×10^{-4}
100	2.54	1.30×10^{-4}	2.74	0.83	1179±16.00	99.33±0.16	1.68×10^{-3}
200	3.60	1.39×10^{-4}	57	0.85	1284±51.00	99.38±0.17	1.39×10^{-3}

Table S2 Electrochemical parameters obtained by fitting EIS results

Concentration (mg/L)	I_0 （A/cm^2）	E_{corr} (V）	η_{PDP} (%)
0	1.64±0.03×10^{-3}	-0.45	——
30	4.75±0.08×10^{-5}	-0.34	97.09±0.067
50	4.05±0.20×10^{-5}	-0.32	97.52±0.112
100	3.91±0.12×10^{-5}	-0.31	97.61±0.082
200	3.12±0.07×10^{-5}	-0.29	98.09±0.039

Table S3 Electrochemical parameters obtained by fitting PDP results

Concentration (mg/L)	Time (h)	R_s ($\Omega\cdot cm^2$)	CPE_f ($S^{n1}\cdot\Omega^{-1}\cdot cm^2$)	R_f ($\Omega\cdot cm^2$)	CPE_{dl}	R_{ct} ($\Omega\cdot cm^2$)	η_{EIS} (%)	σ
0	24	1.32	1.23×10^{-4}	-	0.91	10.99±0.31	—	2.56×10^{-3}
	48	1.06	2.29×10^{-4}	-	0.85	10.46±0.24	—	6.32×10^{-3}
	96	1.10	8.35×10^{-4}	-	0.90	9.13±0.46	—	2.11×10^{-4}
	120	1.11	1.58×10^{-3}	-	0.90	8.01±0.52	—	4.32×10^{-3}
	144	1.12	2.36×10^{-3}	-	0.89	7.15±0.16	—	7.11×10^{-3}
30	24	3.36	3.11×10^{-4}	372.56	0.86	381.6±4.55	97.12±0.034	3.46×10^{-4}
	48	2.51	2.66×10^{-4}	255.34	0.80	256.7±3.80	95.93±0.054	6.31×10^{-4}
	96	1.25	4.75×10^{-4}	174.65	0.78	176.6±5.25	94.29±0.38	1.35×10^{-4}
	120	1.47	1.95×10^{-3}	31.75	0.82	35.61±1.93	77.50±1.28	5.62×10^{-3}
	144	1.35	2.18×10^{-3}	30.11	0.81	34.05±1.97	78.99±1.27	4.52×10^{-4}
200	24	3.59	1.40×10^{-4}	39.82	0.92	1302±21	99.16±0.017	5.21×10^{-3}
	48	2.15	2.12×10^{-4}	611.7	0.85	1232±32	99.15±0.021	6.34×10^{-3}
	96	2.47	2.28×10^{-4}	1061	0.87	1153±4	99.10±0.11	5.44×10^{-4}
	120	2.14	1.95×10^{-4}	1115	0.86	1019±48	99.01±0.23	3.65×10^{-3}
	144	2.72	2.00×10^{-4}	911	0.87	904±53.5	98.89±0.36	7.88×10^{-4}

Table S4 Parameters for EIS measurements with different concentrations of MXene nanosheets.
